# The Role of Diotic and Antiphasic Digits-in-Noise Tests in Predicting Various Types of Hearing Loss in Adolescents

**DOI:** 10.1097/AUD.0000000000001832

**Published:** 2026-05-15

**Authors:** Stefanie N. H. Reijers, Jantien L. Vroegop, Bernd Kremer, Marc P. van der Schroeff

**Affiliations:** 1 Department of Otorhinolaryngology-Head and Neck Surgery, Erasmus University Medical Center, Rotterdam, the Netherlands; 2 The Generation R Study Group, Erasmus University Medical Center, Rotterdam, the Netherlands.

**Keywords:** Digits-in-noise test, Hearing loss, Screening

## Abstract

**Objectives:**

The digits-in-noise (DIN) test is a validated tool for assessing speech reception thresholds in noise and is widely used in hearing screening. The diotic DIN condition presents identical digits and noise to both ears, whereas the antiphasic condition uses phase-inverted digits, which may enhance sensitivity to specific types of hearing loss. This study aimed to evaluate whether the antiphasic DIN condition better predicts various types of hearing loss than the diotic condition, with a particular focus on its value in identifying patterns suggestive of noise-induced hearing loss in adolescents.

**Design:**

This was a cross-sectional study conducted among 2851 adolescents aged 16 to 22 years participating in a population-based cohort study. All participants underwent pure-tone audiometry, tympanometry, and both diotic and antiphasic DIN tests. Hearing loss was categorized based on audiometric criteria into unilateral or bilateral sensorineural hearing loss (SNHL), conductive hearing loss, notched audiograms, and high-frequency hearing loss (HFHL). Logistic regression models were used to examine associations between DIN outcomes and hearing loss categories. Receiver operating characteristic curve analyses were conducted to assess the discriminative ability of the diotic and antiphasic conditions.

**Results:**

Mean DIN speech reception thresholds were −9.0 dB for the diotic condition and −16.1 dB for the antiphasic condition. The antiphasic condition showed stronger correlations with audiometric thresholds and steeper regression slopes, indicating greater sensitivity to hearing loss. It more accurately predicted unilateral SNHL (odds ratio [OR] = 2.48 versus 1.34 for diotic) and conductive hearing loss (OR = 3.42 versus 2.07). Although there was an association with notched audiograms (OR = 1.09), the discriminative ability of the antiphasic condition for detecting this pattern was low (area under the curve [AUC] = 0.515). Predictive values for HFHL were comparable between the two conditions (OR = 1.23 antiphasic versus 1.60 diotic). Overall, the antiphasic DIN condition showed a higher AUC (0.789) for detecting any hearing loss than the diotic condition (0.613).

**Conclusions:**

Both DIN conditions were associated with hearing loss, but the antiphasic condition demonstrated superior sensitivity, particularly for detecting unilateral SNHL and conductive hearing loss. Its association with notched audiograms was statistically significant, yet the low discriminative value limits its practical use in screening for noise-induced hearing loss. For HFHL, both DIN conditions performed similarly, possibly due to the mild degree of hearing loss in most cases. These findings support the use of the antiphasic DIN condition as a more effective screening tool for specific types of hearing loss in adolescents, though its utility for identifying early signs of noise-induced damage remains limited.

## INTRODUCTION

While traditional pure-tone audiometry remains the gold standard for hearing assessment, its widespread use in large-scale screening is often impractical due to time and resource constraints. The digits-in-noise (DIN) test has emerged as a feasible alternative, offering an efficient, app- or web-based method to assess speech recognition in noise (Smits et al. 2004, 2013; de Laat et al. 2016). By presenting digit triplets in speech-shaped noise and determining the speech reception threshold (SRT) in dB signal to noise ratio (SNR) at which a listener can correctly identify 50% of the digits, the DIN provides a practical tool for large-scale screening and early detection (Smits et al. 2004, 2013). Its strong correlation with pure-tone thresholds further supports its utility (Zadeh et al. 2019; Armstrong et al. 2020).

Most DIN tests use a diotic configuration in which identical stimuli are presented to both ears. Although efficient, this setup may lack sensitivity to unilateral or asymmetric sensorineural hearing loss (SNHL), as performance predominantly reflects the better-hearing ear (Potgieter et al. 2018). To address these limitations, an antiphasic modification has been introduced that involves phase-inverted digit presentations between the ears while maintaining in-phase masking noise. This configuration exploits the binaural masking level difference, enhancing sensitivity to specific auditory deficits, such as conductive hearing loss (CHL), and unilateral SNHL (Hirsh 1948; Wilson et al. 1985, 2003; Smits et al. 2016; De Sousa et al. 2020; Polspoel, Moore et al. 2022). The potential benefits of this adaptation extend beyond CHL and SNHL, but its utility for detecting other forms of hearing impairment, such as notches and high-frequency hearing loss (HFHL), remains underexplored.

One of the most rising concerns in adolescent hearing health is noise-induced hearing loss (NIHL), which is becoming increasingly common due to growing exposure to loud environments and the widespread use of personal music devices (Vogel et al. 2014; Paping et al. 2023). Despite these trends, effective screening methods for early-stage hearing loss, particularly notches and HFHL indicative of NIHL, remain lacking (Vlaming et al. 2014). NIHL is typically characterized by a sharp increase in high-frequency thresholds, often presenting as a notch between 3 and 6 kHz (Niskar et al. 2001). This often goes unnoticed due to the dominance of low-frequency perception and its gradual progression, delaying help-seeking behavior. Even mild hearing loss can significantly affect daily life, partic ularly speech understanding in noisy environments, highlighting the need for early detection (Bess et al. 1998; Niclasen et al. 2016; Haile et al. 2021, 2024; le Clercq et al. 2019; Zadeh et al. 2019; Shukla et al. 2020; Overgaard et al. 2021). These mild forms of hearing loss, which may not yet be subjectively noticed by the patient, underscore the necessity of more sensitive and accessible screening tools, particularly in adolescence, when early intervention may reduce long-term consequences (Wake et al. 2004; Teasdale & Sorensen 2007; de Laat et al. 2016).

Despite progress, a review of the literature reveals a notable gap in the understanding of the utility of antiphasic DIN testing for the detection of notches and HFHL. While antiphasic DIN testing has shown promise in detecting CHL and SNHL, its potential benefits for these other forms of hearing loss remain underexplored (De Sousa et al. 2022; Polspoel, Kramer et al. 2022). Given the increasing prevalence of NIHL in adolescents, identifying effective screening tools is critical to reducing the associated long-term consequences (Carroll et al. 2017; le Clercq et al. 2017; Su & Chan 2017; WHO 2024). This study aims to evaluate the predictive value of diotic and antiphasic DIN test configurations for the detection of different types of hearing loss, including notches and HFHL. By directly comparing these two test conditions, this research seeks to clarify the added value of the antiphasic DIN test and contribute to the improvement of subclinical hearing loss detection. This study was conducted in Rotterdam, the Netherlands, at the Erasmus Medical Center.

## MATERIALS AND METHODS

### Study Design and Participants

This research is part of the Generation R study, a prospective cohort initiated in Rotterdam following individuals from prenatal stages into adulthood Kooijman et al. (2016). Pregnant women with expected delivery dates between April 2002 and January 2006 were recruited (n = 9778). Data were collected via questionnaires, interviews, and measurements during a 4 hr visit to the Erasmus Medical Center. For the present analysis, we included adolescents from the original cohort who were invited for hearing assessments at age 18 years, whose actual ages at testing ranged from 16 years 2 months to 21 years 9 months (data collection: October 2020 to May 2024). Of the invited participants, 2851 adolescents successfully completed pure-tone audiometry, tympanometry, and DIN testing, all conducted on the same day. Individuals were excluded if testing was incomplete due to time limitations, technical problems, or personal circumstances. The sample included individuals with normal hearing, unilateral and bilateral SNHL, CHL, notches, and HFHL. Ethical approval was granted by the Medical Ethical Committee of Erasmus Medical Center Rotterdam, and written consent was obtained.

### Pure-Tone Audiometry

Pure-tone audiometry was conducted in a soundproof booth meeting ISO standard 8253-1. A Decos audiometer (version 210.2.6 with AudioNigma interface) with TDH-39P headphones was used. Thresholds at 0.5, 1, 2, 3, 4, 6, and 8 kHz were measured using the ISO ascending method, identifying the level at which tones were heard in two of three trials. Each ear was tested alternately, and bone conduction was excluded due to time constraints. Testing was performed by dedicated research assistants who were trained by a member of the Speech and Hearing Center.

### Speech-in-Noise Audiometry

The Dutch DIN test was used, presenting digit triplets binaurally with steady-state noise at 70 dB SPL. The intensity of the speech signal was dynamically adjusted using an adaptive procedure to determine the SNR corresponding to a 50% correct response rate, known as the DIN SRT in noise, as outlined by Smits et al. (2004). A series of 24 digit triplets was presented, starting with the initial triplet at −10 dB SNR and then increasing by 4 dB until it was correctly perceived for the first time. The adaptive process involved a fixed step size, with a reduction of 2 dB in intensity for the next triplet following a correct response, while an incorrect response prompted an increase of 2 dB in intensity for the subsequent triplet. On completion of the 24 triplets, the DIN SRT (dB) and the SD were automatically calculated by the computer over the final 20 triplets. This assessment included two conditions: Condition 1 (S_0_N_0_), where the signal and noise were identical in both ears (diotic), and Condition 2 (S_0_N_π_), where the signal in one ear was out of phase with the signal in the other ear (antiphasic).

The DIN test was conducted in a soundproof booth using a prerecorded male voice. A single speaker was used throughout the testing. The digits were limited to 0 to 9, and all triplets were prerecorded, with amplitude normalization applied to ensure consistent loudness across digits, including bisyllabic ones. The two test conditions were administered consecutively, with the order randomized across participants. Participants were given standardized instructions for each condition to repeat the three digits they heard, but were blinded to the difference between diotic and antiphasic testing. Responses were provided verbally and scored by the administering researcher. The entire test duration per participant was approximately 7 min.

### Tympanometry

Tympanometry was performed to assess middle ear function, unless contraindicated by conditions such as excessive wax, ear discharge, or recent surgery. Using an Interacoustics AT235h tympanometer with a 226-Hz probe tone, the assessment measured ear canal volume, middle ear pressure, and compliance through a pressure sweep. Canal volumes <0.3 mL were excluded. Tympanograms were classified as type A (normal: compliance ≥0.25 mL, pressure −100 to +100 daPa) or type B/C (possible pathology) (Jerger 1970).

### Definitions of Hearing Loss Categories

Normal hearing was defined as a mean threshold of ≤15 dB HL in both ears (n = 2342), regardless of tympanogram. Unilateral or asymmetric SNHL was defined as one ear having a mean threshold of >15 dB HL in either the low-frequencies (0.5, 1, 2 kHz) or high-frequencies (3, 4, 6 kHz) with a type A tympanogram, while the other ear had a mean threshold of ≤15 dB HL (n = 44). Bilateral SNHL was characterized by a mean threshold of >15 dB HL in either the low- or high-frequencies in both ears, with type A tympanograms (n = 7). CHL was defined as a mean threshold of >15 dB HL and a type B or C tympanogram in one or both ears (n = 27) (le Clercq et al. 2017). A notch (n = 197) was defined according to the criteria of (Niskar et al. 1998), which include: (1) thresholds of ≤15 dB HL at 0.5 and 1 kHz; (2) the poorest threshold at 3, 4, or 6 kHz being at least 15 dB or worse than the poorest threshold at 0.5 and 1 kHz; and (3) a threshold at 8 kHz that is at least 10 dB better than the poorest threshold at 3, 4, or 6 kHz (Niskar et al. 2001). HFHL (n = 234) was defined as (1) hearing thresholds of ≤15 dB HL at 0.5 and 1 kHz, and (2) an average threshold at 3, 4, 6, and 8 kHz of >15 dB HL. A notch or HFHL was considered present in conjunction with a type A tympanogram. Hearing categories were based on mean pure-tone average (PTA) of 0.5, 1, 2, 3, 4, and 6 kHz, and categorized as normal (0 to 15 dB HL), slight to mild (16 to 40 dB HL), moderate (41 to 55 dB HL), and severe to profound (56 to 120 dB HL), in accordance with the American Speech-Language-Hearing Association guidelines and in line with other prevalence studies (Clark 1981; le Clercq et al. 2017).

### Statistical Analysis

Mean DIN performance was calculated. Binary logistic regression assessed associations between SNR and hearing loss. Multinomial logistic regression examined predictive value for hearing categories (bilateral/unilateral SNHL, CHL, notches, HFHL). Receiver operating characteristic (ROC) curves and AUCs evaluated discriminative ability. As an additional analysis, we performed one-way ANOVAs with post-hoc Tukey HSD tests to compare diotic and antiphasic DIN SRTs across hearing categories. These results are provided as Supplemental Digital Content, https://links.lww.com/EANDH/B898. Analyses were conducted in R version 4.3.2.

## RESULTS

**Fig. 1.**
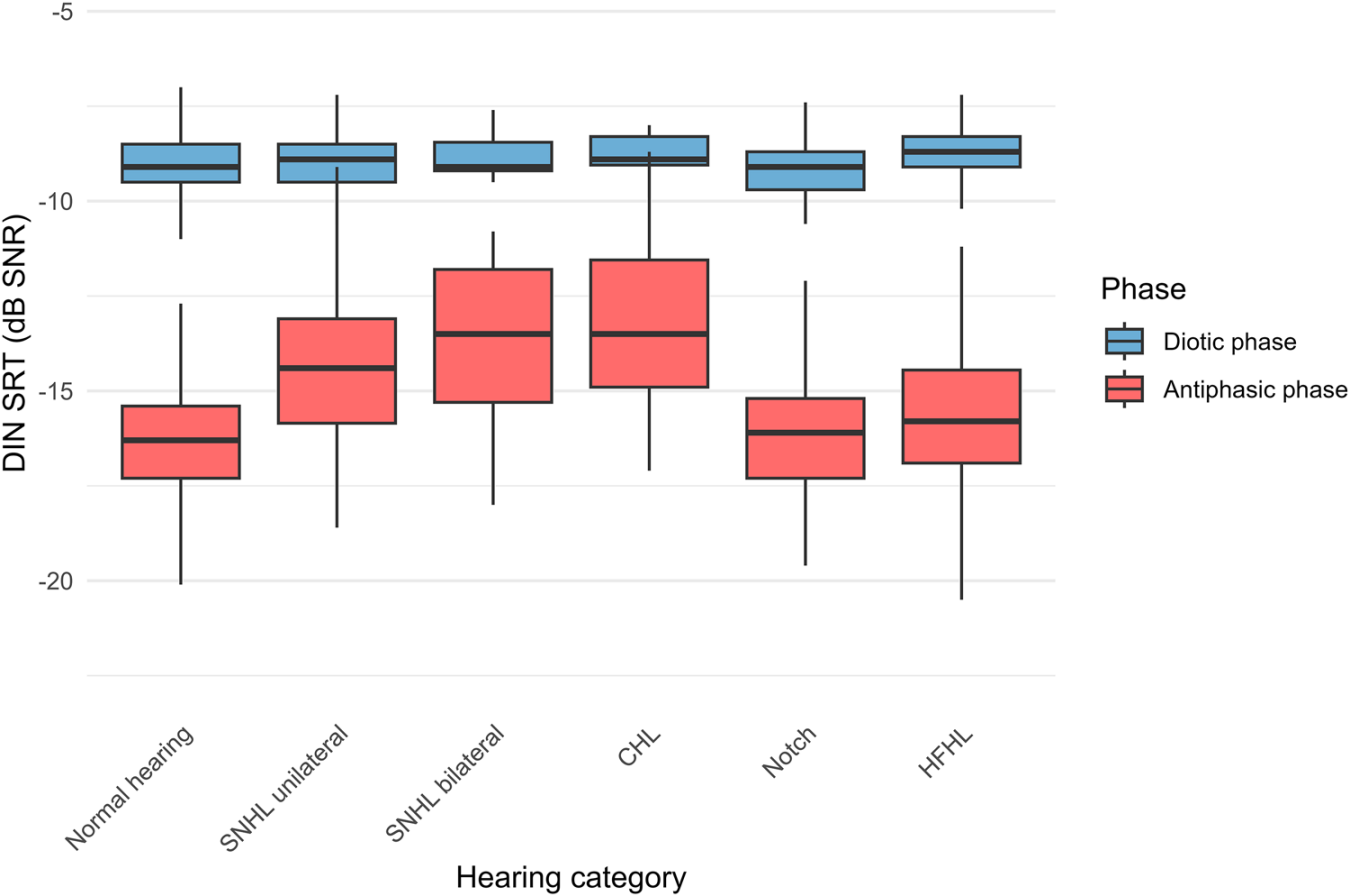
Mean DIN SRT for the diotic and antiphasic condition in different hearing categories. Boxplots showing the diotic and antiphasic conditions across different hearing categories. CHL indicates conductive hearing loss; dB, decibel; DIN, digits-in-noise; HFHL, high-frequency hearing loss; PTA, pure-tone average; SNHL, sensorineural hearing loss; SNR, signal to noise ratio.

A total of 2851 adolescents with complete data for pure-tone audiometry, the diotic and antiphasic tests, and tympanometry were included in the analysis. Of these, 1510 (53.0%) were girls, 1337 (46.9%) were boys, and 4 (0.1%) did not identify as a girl or boy. The mean age was 18.4 years (SD = 8 months; range 16 years and 2 months to 21 years and 9 months). The mean PTA (0.5, 1, 2, 3, 4, and 6 kHz) for the whole cohort was 5.5 dB HL (SD = 4.3) for the right ear and 6.1 dB HL (SD = 5.1) for the left ear. The mean DIN SRT for the whole cohort was −9.0 dB (SD = 0.9) in the diotic condition, and −16.1 dB (SD = 1.9) in the antiphasic condition. The different diotic and antiphasic DIN SRTs according to degree of hearing are shown in Table 1 and Figure 1.

**TABLE 1.** Diotic and antiphasic DIN SRTs according to the degree of hearing in the poorer ear and hearing categories

Degree of Hearing		Normal (0–15 dB HL)	Slight to Mild (16–40 dB HL)	Moderate (41–55 dB HL)	Severe to Profound (56–120 dB HL)
Normal hearing and bilateral SNHL (n = 2349)	Diotic (n) Mean SRT (SD)	2342 −9.0 (0.8)	6 −8.3 (1.0)	1 −6.8 (1.4)	
	Antiphasic (n) Mean SRT (SD)	2342 −16.2 (1.8)	6 −14.8 (2.9)	1 −9.3 (1.9)	
Unilateral SNHL (n = 44)	Diotic (n) Mean SRT (SD)		42 −8.8 (0.8)	1 −9.1 (2.3)	1 −8.1 (1.5)
	Antiphasic (n) Mean SRT (SD)		42 −15.3 (2.2)	1 −12.7 (2.6)	1 −8.9 (1.7)
CHL (n = 27)	Diotic (n) Mean SRT (SD)		23 −8.1 (2.1)	3 −6.8 (2.1)	1 −8.5 (1.6)
	Antiphasic (n) Mean SRT (SD)		23 −13.6 (2.1)	3 −9.8 (1.9)	1 −9.7 (1.6)
Notch (n = 197)	Diotic (n) Mean SRT (SD)	186 −9.0 (0.7)	10 −8.4 (1.8)	1 −8.1 (1.5)	
	Antiphasic (n) Mean SRT (SD)	186 −16.0 (2.0)	10 −14.9 (2.2)	1 −8.9 (1.7)	
HFHL (n = 234)	Diotic (n) Mean SRT (SD)		223 −8.6 (0.9)	9 −7.9 (1.8)	2 −7.7 (1.6)
	Antiphasic (n) Mean SRT (SD)		223 −15.3 (2.1)	9 −11.8 (2.2)	2 −9.3 (1.7)

CHL, conductive hearing loss; HFHL, high-frequency hearing loss; SNHL, sensorineural hearing loss; SRT indicates speech reception threshold.

**Fig. 2.**
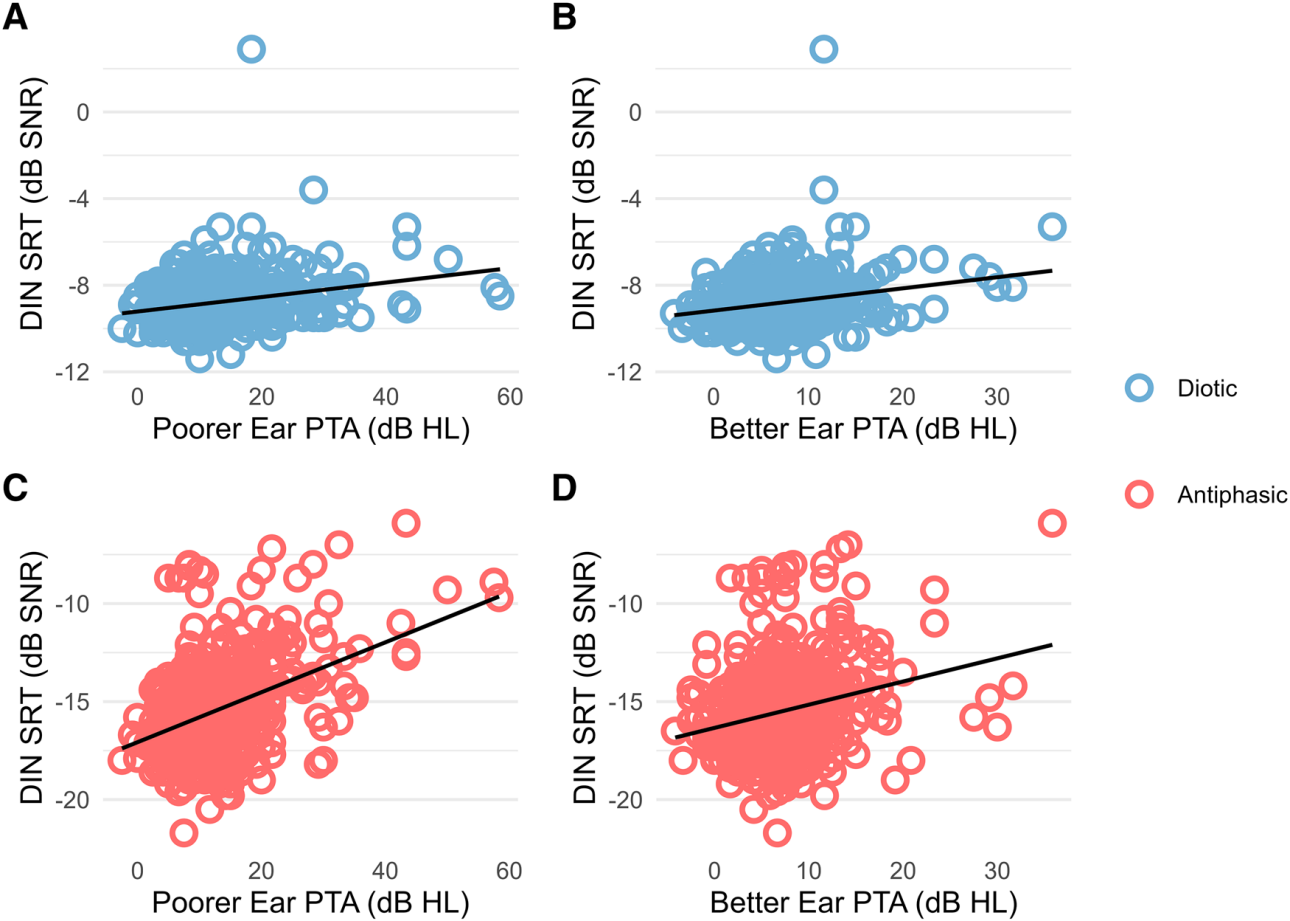
Correlations of the diotic DIN and antiphasic DIN to poorer and better ear PTA. Correlations of the diotic DIN (A: poorer ear, B: better ear) and antiphasic DIN (C: poorer ear, D: better ear) with PTA. DIN indicates digits-in-noise; PTA, pure-tone average.

**Fig. 3.**
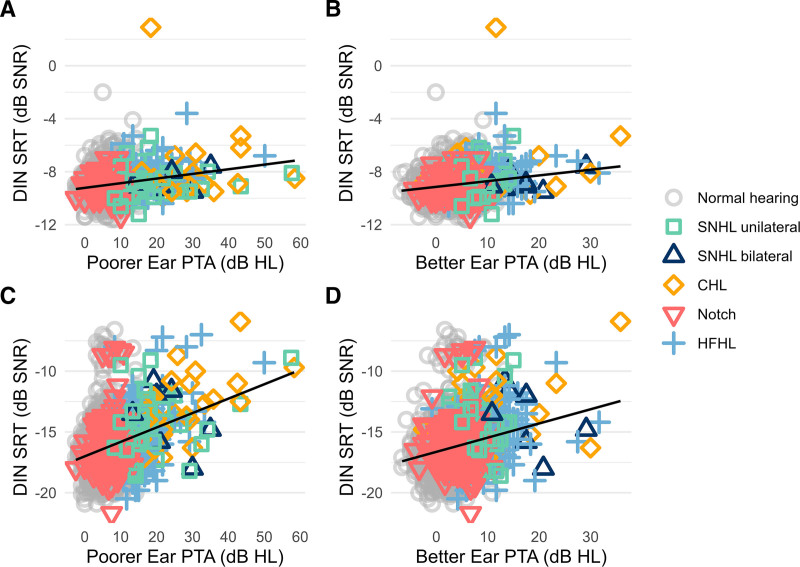
Correlations between the diotic DIN and antiphasic DIN with the poorer and better ear PTA across different hearing categories. Correlations of the diotic DIN (A: poorer ear, B: better ear) and antiphasic DIN (C: poorer ear, D: better ear) with PTA. CHL indicates conductive hearing loss; dB, decibel; DIN, digits-in-noise; HFHL, high-frequency hearing loss; PTA, pure-tone average; SNHL, sensorineural hearing loss; SNR, signal to noise ratio.

In the total cohort, both poorer ear PTA and better ear PTA (dB HL) were significantly correlated with diotic and antiphasic DIN SRT (*p* < 0.001) (Figs. 2 and 3). However, the correlation was stronger for antiphasic DIN SRT compared with diotic DIN SRT (poorer ear PTA: *r* = 0.82 versus *r* = 0.44; better ear PTA: *r* = 0.47 versus *r* = 0.30). A statistically significant difference was found between the correlations for diotic and antiphasic conditions for poorer ear PTA (Z = −4.55, *p* = 5.37 × 10^−^6), but no significant difference was found for better ear PTA (*Z* = −1.89, *p* = 0.058). Regression analyses showed that the slope for the diotic condition was 0.035 for the poorer ear PTA and 0.043 for the better ear PTA, whereas the slope for the antiphasic condition was significantly steeper at 0.119 for the poorer ear PTA and 0.117 for the better ear PTA. The difference in slopes was statistically significant for both poorer ear PTA (*Z* = 10.99, *p* < 0.001) and better ear PTA (*Z* = 7.28, *p* < 0.001). In participants with a notch, poorer ear PTA was not significantly correlated with diotic or antiphasic DIN SRT (*p* = 0.296 and *p* = 0.105, respectively). However, in those with HFHL, significant correlations were found between poorer ear PTA and both diotic (*r* = 0.25, *p* < 0.001) and antiphasic DIN SRT (*r* = 0.32, *p* < 0.001), with a stronger correlation for the antiphasic condition.

Multinomial logistic regression analyses were conducted to examine the predictive value of the diotic and antiphasic DIN test conditions for different hearing loss categories. In the diotic condition, the odds ratio (OR) for unilateral SNHL was 1.34 (95% CI: 1.14 to 1.57, *p* < 0.001), while in the antiphasic condition it was 2.48 (95% CI: 1.29 to 3.67, *p* < 0.05). For bilateral SNHL, the diotic condition showed an OR of 1.43 (95% CI: 0.92 to 2.23, *p* < 0.05), and the antiphasic condition 1.57 (95% CI: 1.13 to 2.17, *p* < 0.05). The antiphasic condition also predicted CHL more strongly (OR: 3.42, 95% CI: 2.61 to 4.52, *p* < 0.001) than the diotic condition (OR: 2.07, 95% CI: 1.46 to 2.92, *p* < 0.001). For notches, the diotic condition showed an OR of 0.87 (95% CI: 0.74 to 1.03, *p* = 0.10), indicating a non-significant trend toward prediction. However, the antiphasic condition showed a significant association with notches, with an OR of 1.09 (95% CI: 1.01 to 1.19, *p* < 0.05). For HFHL, the diotic condition had an OR of 1.60 (95% CI: 1.36 to 1.89, *p* < 0.001), and the antiphasic condition 1.23 (95% CI: 1.07 to 1.42, *p* < 0.01). In a sub-analysis examining unilateral (n = 190) and bilateral (n = 44) HFHL, both conditions significantly predicted unilateral HFHL (*p* < 0.01), with ORs of 1.12 and 1.27, respectively. Only the diotic condition was significant for bilateral HFHL (*p* < 0.01, OR: 1.45).

**Fig. 4.**
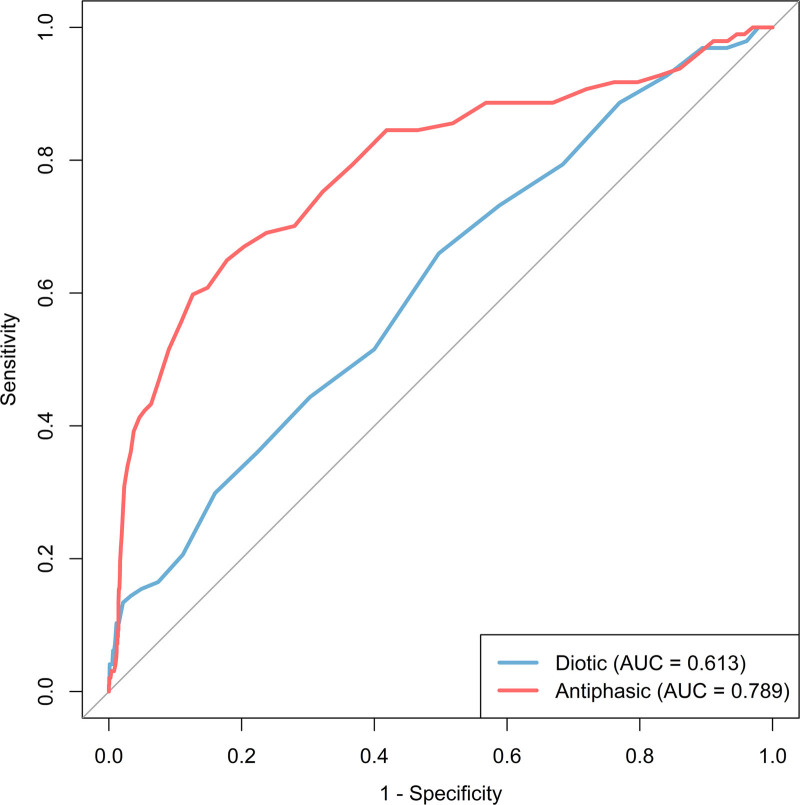
Comparison of ROC curves of diotic and antiphasic condition for poorer ear PTA >15 dB HL. ROC curves presenting test characteristics of the antiphasic DIN and diotic for detecting poorer ear PTA >15 dB HL. AUC indicates area under the curve; dB, decibel; DIN, digits-in-noise; HL, hearing level; PTA, pure-tone average; ROC, receiver operating characteristics.

**Fig. 5.**
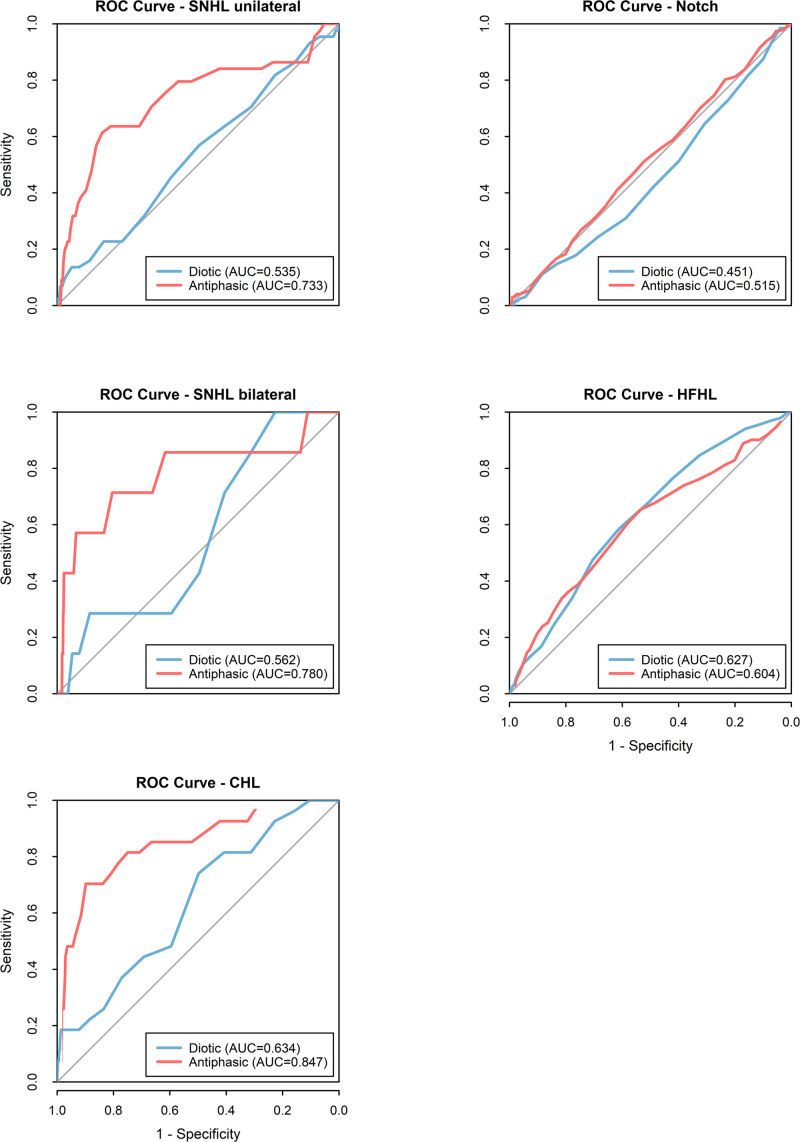
Comparison of ROC curves of diotic and antiphasic condition for different types of hearing loss. ROC curves showing the diagnostic performance of the diotic and antiphasic DIN conditions for detecting different types of hearing loss: unilateral SNHL, bilateral SNHL, CHL, notch, and HFHL. CHL indicates conductive hearing loss; DIN, digits-in-noise; HFHL, high-frequency hearing loss; ROC, receiver operating characteristic; SNHL, sensorineural hearing loss.

ROC curve analysis assessed model performance (Fig. 4). The AUC for the diotic condition was 0.613, indicating moderate discriminatory ability. For the antiphasic condition, the AUC was 0.789, reflecting a stronger predictive value for hearing loss. ROC curves for the specific hearing loss categories, including notches and HFHL, are shown in Figure 5. The AUC values for notches were 0.451 (diotic) and 0.515 (antiphasic), indicating limited predictive ability. For HFHL, the AUC values were 0.627 (diotic) and 0.604 (antiphasic), indicating moderate predictive value. Table 1 in Supplemental Digital Content, https://links.lww.com/EANDH/B898, provides the sensitivity, specificity, and optimal SRT thresholds for diotic and antiphasic DIN conditions for each hearing loss category.

As a supplementary analysis (Table 2 in Supplemental Digital Content, https://links.lww.com/EANDH/B898), DIN SRTs were compared across hearing categories within each test condition. For the diotic phase, CHL and HFHL groups showed higher SRTs than normal hearing, whereas differences for SNHL (unilateral or bilateral) and small notches were minimal. In the antiphasic phase, CHL, SNHL (unilateral and bilateral), and HFHL groups showed more pronounced differences from normal hearing, while small notches again showed limited differentiation. These results suggest that the antiphasic DIN better distinguishes between hearing loss categories, although subtle deficits, such as small notches, remain difficult to detect.

## DISCUSSION

This study investigated the predictive value of the diotic and antiphasic conditions of the DIN test for detecting different types of hearing loss in adolescents aged 16 to 22 years. The antiphasic condition showed stronger correlations and steeper slopes for both the poorer and better ear PTAs, as well as a higher discriminatory ability (AUC = 0.789) compared with the diotic condition in predicting the presence of hearing loss (>15 dB HL). Additionally, the antiphasic DIN test showed a stronger predictive value for unilateral and bilateral SNHL, CHL, and notches. However, for HFHL, both DIN tests were significantly associated, showing comparable predictive performance.

Our study examined the ability of the antiphasic DIN test to detect notches indicative of NIHL, a growing concern in adolescents due to increased noise exposure from personal music devices and other sources (Vogel et al. 2014; Paping et al. 2023). While the antiphasic condition showed a slightly better predictive value for notches (OR = 1.09, 95% CI: 1.01 to 1.19), the AUC value was too low for practical application in our cohort. It is important to note that these analyses address different questions, within-group variability versus between-group classification, which explains the apparent discrepancy. This suggests that the antiphasic DIN has limited ability to distinguish individuals with small, early-stage notches, and its discriminatory power for detecting these subtle changes remains modest. A possible explanation is that many adolescents in our cohort with a notch still had an average PTA below 16 dB HL, which is within the normal hearing range. The DIN may still hold value for detecting more pronounced notches or established NIHL. This warrants further investigation to better understand the relationship between notch patterns and hearing thresholds in this population.

When comparing our results with the existing literature, the stronger association of the antiphasic DIN test with SNHL and CHL is consistent with previous studies that emphasized the antiphasic condition’s enhanced sensitivity. De Sousa et al. (2020) and Polspoel et al. (2022) demonstrated that the antiphasic condition better captures binaural auditory deficits due to its reliance on the binaural masking level difference. It is interesting that previous studies have demonstrated significant correlations between HFHL (4 to 8 kHz) and speech perception in noise, as speech-in-noise difficulties are often one of the first noticeable issues for individuals with HFHL (Jansen et al. 2013; Vlaming et al. 2014). Given this established relationship, the absence of strong findings for HFHL in our study is particularly striking, especially considering the established links between HFHL and speech-in-noise difficulties. One possible explanation is that the HFHL losses in our sample were still relatively mild, potentially limiting the strength of these correlations. Even when focusing solely on unilateral HFHL cases, there was no clear advantage for the antiphasic condition, as initially hypothesized. When looking at the bilateral HFHL cases, only the diotic condition remained a significant predictor.

The design of the DIN test itself may also explain the comparable predictive performance of both test conditions for HFHL. The Dutch DIN test primarily targets speech-in-noise recognition at frequencies between 500 and 4000 Hz, which limits its sensitivity to HFHL, particularly at frequencies above 6 kHz. Consequently, both the diotic and antiphasic conditions may have been less sensitive to HFHL, especially in cases where deficits occur above the 8 kHz threshold. The antiphasic signal enhances binaural masking release mainly in the frequency range tested, which may explain why it does not confer additional predictive value for higher-frequency losses. Škerková et al. (2021) emphasize the importance of extended high-frequency audiometry in detecting early stages of HFHL, as well as the crucial role of high-frequency hearing in speech perception in noise. Although some recent studies suggest that frequencies above 8 kHz may contribute to speech recognition in noise, most of this evidence comes from research in individuals with normal hearing (Polspoel et al. 2022). The question remains whether these factors already contribute to the DIN score in our cohort.

Previous research (Zadeh et al. 2021) has suggested that modifying the DIN test by incorporating a low-pass filtered masker could enhance sensitivity to NIHL, but this does not necessarily imply that extending the frequency range of pure-tone audiometry would lead to stronger correlations with the DIN score. Leensen et al. (2011) demonstrate that this masking technique enhances sensitivity to NIHL, potentially improving the detection of HFHL within the DIN test. Further research is needed to assess the impact of such modifications. Including high-frequency measures in future studies could provide a better understanding of their relationship with speech recognition, although evidence for this is still in early stages.

A limitation of our study is that bone conduction thresholds were not measured due to time and personnel constraints. Consequently, CHL was defined solely based on tympanometry. As middle ear dysfunction does not always correspond to a conductive component on audiometry, this approach may have led to misclassification and could affect the accuracy of CHL detection.

Our results suggest that the antiphasic DIN test could complement existing hearing screening methods, particularly for detecting SNHL and CHL. While the antiphasic condition showed a slightly higher predictive value for notches, its overall ability to discriminate between individuals with and without notches was limited, making it unlikely to be useful for this purpose in practice. This warrants further investigation into the relationship between notches and overall hearing thresholds. For HFHL, both conditions remain effective, but their utility could potentially be enhanced by focusing on more severe hearing losses and incorporating comprehensive high-frequency audiometry. Future research should explore the use of the antiphasic DIN test in more diverse populations and assess its effectiveness in individuals with more pronounced hearing deviations.

## CONCLUSIONS

In this large cohort, both diotic and antiphasic DIN test conditions were able to predict hearing loss, with the antiphasic condition demonstrating greater sensitivity, particularly for unilateral SNHL and CHL. It showed stronger associations with pure-tone audiometry and had a higher discriminative ability (AUC = 0.789), confirming its potential as a screening tool. However, its predictive value for notches was limited, suggesting that it may not be suitable for detecting NIHL in adolescents. In addition, both conditions performed similarly for HFHL, likely due to the predominance of mild losses in the cohort. Future research should explore its applicability in populations with more pronounced hearing deviations and assess whether integrating extended high-frequency audiometry could improve its diagnostic utility.

## ACKNOWLEDGMENTS

The authors thank Teun van Immerzeel for his contribution to programming the Digits-In-Noise (DIN) test. His work has played an important role in the execution of the study.

## Supplementary Material
