# Supplementary material for: The Role of Diotic and Antiphasic Digits-in-Noise Tests in Predicting Various Types of Hearing Loss in Adolescents
Source: Ear Hear. 2026 May 15;47(5):1380–7. doi: 10.1097/AUD.0000000000001832 (PMC13465695; doi:10.1097/AUD.0000000000001832)
Supplement: Supplementary file 1 [file aud-47-1380-s001.pdf]

Supplementary Material

Table 1. Sensitivity, specificity, and optimal SRT cut-off values for diotic and antiphasic DIN conditions across hearing loss categories

DIN_condition	Hearing_loss	threshold	sensitivity	specificity
Diotic	SNHL unilateral	-7.7	0.136363636	0.947630923
Antiphasic	SNHL unilateral	-14.7	0.613636364	0.84004275
Diotic	SNHL bilateral	-9.6	1	0.226793249
Antiphasic	SNHL bilateral	-14.9	0.714285714	0.804149086
Diotic	CHL	-9	0.740740741	0.498229462
Antiphasic	CHL	-14.1	0.703703704	0.898371105
Diotic	Notch	-10.3	0.984771574	0.039939714
Antiphasic	Notch	-17.4	0.802030457	0.235116805
Diotic	HFHL	-8.8	0.585470085	0.611387084
Antiphasic	HFHL	-16.2	0.653846154	0.534963699

Table 2. Pairwise comparisons of diotic and antiphasic DIN SRTs across hearing loss categories

Condition	Comparison	diff	lwr	upr	p adj
Diotic	SNHL unilateral-Normal hearing	0.209251999	-0.163367309	0.581871307	0.598
Diotic	SNHL bilateral-Normal hearing	0.216719532	-0.710214787	1.14365385	0.986
Diotic	CHL-Normal hearing	0.954285669	0.480308829	1.42826251	1.54E-07
Diotic	Notch-Normal hearing	-0.090967198	-0.272625274	0.090690878	0.71
Diotic	HFHL-Normal hearing	0.373801339	0.205912636	0.541690042	2.34E-09
Diotic	SNHL bilateral-SNHL unilateral	0.007467532	-0.98899196	1.003927024	1
Diotic	CHL-SNHL unilateral	0.74503367	0.146386584	1.343680756	0.00527
Diotic	Notch-SNHL unilateral	-0.300219197	-0.708537633	0.108099238	0.289
Diotic	HFHL-SNHL unilateral	0.16454934	-0.237832202	0.566930881	0.853
Diotic	CHL-SNHL bilateral	0.737566138	-0.301058153	1.776190428	0.328
Diotic	Notch-SNHL bilateral	-0.30768673	-1.249539174	0.634165715	0.938
Diotic	HFHL-SNHL bilateral	0.157081807	-0.78221207	1.096375684	0.997
Diotic	Notch-CHL	-1.045252867	-1.547779326	-0.542726409	4.90E-08
Diotic	HFHL-CHL	-0.58048433	-1.078198909	-0.082769752	0.0115
Diotic	HFHL-Notch	0.464768537	0.227987176	0.701549897	3.55E-07
Antiphasic	SNHL unilateral-Normal hearing	1.904285382	1.119499826	2.689070937	0
Antiphasic	SNHL bilateral-	2.504934732	0.552688434	4.45718103	0.00351

	Normal hearing				
Antiphasic	CHL-Normal hearing	3.246204573	2.247946646	4.244462501	0
Antiphasic	Notch-Normal hearing	0.261715008	-0.120880932	0.644310948	0.371
Antiphasic	HFHL-Normal hearing	0.750905428	0.39730961	1.104501246	2.22E-08
Antiphasic	SNHL bilateral-SNHL unilateral	0.600649351	-1.49802616	2.699324861	0.965
Antiphasic	CHL-SNHL unilateral	1.341919192	0.081089235	2.602749149	0.0292
Antiphasic	Notch-SNHL unilateral	-1.642570374	-2.502543015	-0.782597733	8.30E-07
Antiphasic	HFHL-SNHL unilateral	-1.153379953	-2.00084871	-0.305911197	0.00149
Antiphasic	CHL-SNHL bilateral	0.741269841	-1.446210312	2.928749995	0.929
Antiphasic	Notch-SNHL bilateral	-2.243219724	-4.226885569	-0.25955388	0.0161
Antiphasic	HFHL-SNHL bilateral	-1.754029304	-3.732306467	0.224247859	0.116
Antiphasic	Notch-CHL	-2.984489566	-4.042876766	-1.926102366	0
Antiphasic	HFHL-CHL	-2.495299145	-3.543551889	-1.447046401	0
Antiphasic	HFHL-Notch	0.48919042	-0.009502449	0.98788329	0.0581
